# Humidification during Mechanical Ventilation in the Adult Patient

**DOI:** 10.1155/2014/715434

**Published:** 2014-06-25

**Authors:** Haitham S. Al Ashry, Ariel M. Modrykamien

**Affiliations:** ^1^Division of Internal Medicine, Department of Medicine, Creighton University Medical Center, Omaha, NE 68131, USA; ^2^Respiratory Care and Pulmonary Function Laboratory, Division of Pulmonary and Critical Care Medicine, Baylor University Medical Center, 3600 Gaston Avenue, Wadley Tower 1155, Dallas, TX 75246, USA

## Abstract

Humidification of inhaled gases has been standard of care in mechanical ventilation for a long period of time. More than a century ago, a variety of reports described important airway damage by applying dry gases during artificial ventilation. Consequently, respiratory care providers have been utilizing external humidifiers to compensate for the lack of natural humidification mechanisms when the upper airway is bypassed. Particularly, active and passive humidification devices have rapidly evolved. Sophisticated systems composed of reservoirs, wires, heating devices, and other elements have become part of our usual armamentarium in the intensive care unit. Therefore, basic knowledge of the mechanisms of action of each of these devices, as well as their advantages and disadvantages, becomes a necessity for the respiratory care and intensive care practitioner. In this paper, we review current methods of airway humidification during invasive mechanical ventilation of adult patients. We describe a variety of devices and describe the eventual applications according to specific clinical conditions.

## 1. Introduction

In 1871, Friedrich Trendelenburg described the first endotracheal intubation for administration of general anesthesia [1]. Since then, there has been a growing body of literature addressing the effect of dry gases on respiratory tract of intubated patients. In fact, a study on eighteen patients undergoing general anesthesia demonstrated that after three hours of exposure to dry anesthetic gas, respiratory epithelial cells had 39% ciliar damage, 39% cytoplasmic changes, and 48% nuclear changes [2]. Later on, other authors examined the effect of dry gas on mucous flow in dogs anaesthetized for heart-lung bypass operations. In the group exposed to dry gas, mucous flow had reduced clearance velocity compared to the group that inhaled completely humidified gas [3]. Over the course of the years, a large body of literature revealed the unfavorable effects of inadequate humidification on the respiratory tract [4–10]. Consequently, humidification during invasive mechanical ventilation is currently an accepted standard of care [11].

In this review, we aim to describe the basic principles of airway humidification on mechanically ventilated patients, the most commonly used humidifier devices, and the proper selection of humidifiers according to the clinical condition.

## 2. Physiological Airway Control of Heat and Humidity

Humidity is the amount of water in vaporous state contained in a gas. Humidity is usually characterized in terms of absolute or relative humidity. Absolute humidity (AH) is the weight of water present in a given volume of gas and it is usually expressed in mg/L. Relative humidity (RH) is the ratio of the actual weight of water vapor (AH) relative to the gas capacity to keep water at a specific temperature. Whenever the amount of gas contained in a sample is equal to its water vapor capacity, the RH is 100% and the gas is completely saturated. It is important to understand that water vapor capacity of a sample will increase exponentially to the temperature [3]. Therefore, if the absolute humidity remains constant, RH will decrease whenever the temperature increases (because the denominator increases), and RH will increase when the temperature decreases (because the capacity to hold water vapor decreases). In the later situation, as the content of water in the gas exceeds its holding capacity, water will condensate into liquid droplets. This situation becomes particularly relevant to mechanically ventilated patients, as liquid water has a tendency to accumulate in the lower point of the tubing, increasing resistance to gas delivery. At the level of the sea, the capacity of gas to hold water at body temperature and pressure saturated (BTPS) is 43.9 mg of water per liter of gas.  Table 1 shows humidity requirements for gas delivery at different anatomic sites in the airway [12].

Heat and moisture exchange is one of the most important functions of the respiratory system. The connective tissue of the nose is characterized by a rich vascular system of numerous and thin walled veins. This system is responsible for warming the inspired air to increase its humidity carrying capacity. As the inspired air goes down the respiratory tract, it reaches a point at which its temperature is 37°C and its relative humidity is 100%. This point is known as the isothermic saturation boundary (ISB), and it is usually located 5 cm below the carina [13]. The respiratory mucosa is lined by pseudostratified columnar ciliated epithelium and with numerous goblet cells. These cells, as well as submucosal glands underneath the epithelium, are responsible for maintaining the mucous layer that serves as a trap for pathogens and as an interface for humidity exchange. At the level of the terminal bronchioles, the epithelium turns into a simple cuboidal type with minimal goblet cells and scarce submucosal glands. Hence, the capacity of these airways to carry on the same level of humidification maintained by upper airways is limited [14]. After endotracheal intubation, as the upper airway loses its capacity to heat and moisture inhaled gas, the ISB is shifted down the respiratory tract. This imposes a burden on the lower respiratory tract, as it is not well prepared for the humidification process. Consequently, delivery of partially cold and dry medical gases brings about potential damage to the respiratory epithelium, manifested by increased work of breathing, atelectasis, thick and dehydrated secretions, and cough and/or bronchospasm [15]. Notably, there are other factors that may shift the ISB distally producing the same effects, such as mouth breathing, cold and dry air breathing, and/or high minute ventilation. In fact, inhalation of large volumes of cold air during exercise is thought to be the inciting event of exercise-induced asthma [16].

During the exhalation process, the expired gas transfers heat back to the upper airway mucosa. As the airway temperature decreases, the capacity to hold water also decreases. Therefore, condensed water is reabsorbed by the mucosa, recovering its hydration. Importantly, in periods of cold weather, the amount of water condensation may exceed the mucosal capacity to accept water. Therefore, the remaining water accumulates in the upper airway with consequent rhinorrhea.

In order to avoid the aforementioned consequences associated with lack of humidification in mechanically ventilated patients, a variety of devices (humidifiers) have been introduced in clinical practice. In the following paragraphs, we describe current types of humidifiers utilized in mechanical ventilation.

## 3. Types of Humidifiers

Humidifiers are devices that add molecules of water to gas. They are classified as active or passive based on the presence of external sources of heat and water (active humidifiers), or the utilization of patients' own temperature and hydration to achieve humidification in successive breaths (passive humidifiers).

### 3.1. Active Humidifiers

**Figure 1:**
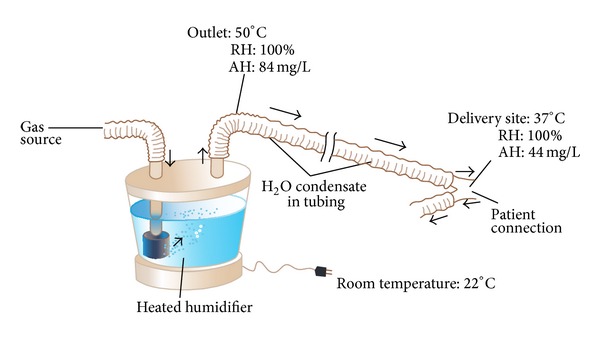
Heated humidifier and condensation, adapted from* Egan's Fundamentals of Respiratory Care, *10th edition, St. Louis: Mosby-Elsevier; 2012: 1424 [17].

**Figure 2:**
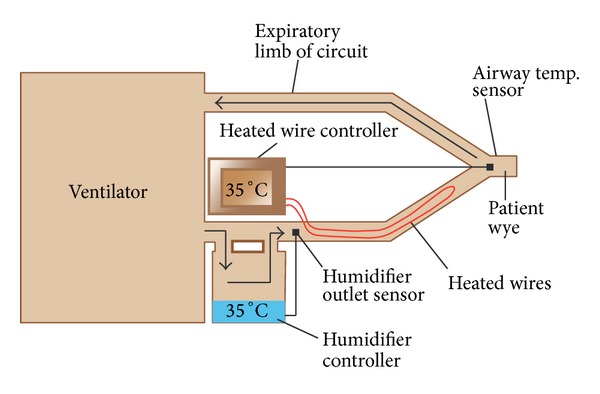
Humidifier with heated wire in the inspiratory limb, adapted from* Egan's Fundamentals of Respiratory Care, *10th edition, St. Louis: Mosby-Elsevier; 2012: 1424 [17].

Active humidifiers act by allowing air passage inside a heated water reservoir. These devices are placed in the inspiratory limb of the ventilator circuit, proximal to the ventilator. After the air is loaded with water vapor in the reservoir, it travels along the inspiratory limb to the patient's airway. As condensation of water vapor may accumulate as the surrounding temperature of the inspiratory limb decreases, these systems are used with the addition of water traps, which require frequent evacuation to avoid risk of contamination of the circuit.  Figure 1 shows a diagram of a heated humidifier that operates at 50°C to achieve an AH of 84 mg/L at the side of the humidifier but achieves only an AH of 44 mg/L due to significant condensate in the tubing [17]. Due to the aforementioned shortcoming, heated humidifiers are usually supplied with heated wires (HWH) along the inspiratory limb to minimize this problem. These humidifiers have sensors at the outlet of the humidifier and at the Y-piece, near the patient. These sensors work in a closed-loop fashion, providing continuous feedback to a central regulator to maintain the desired temperature at the distal level (Y-piece). When the actual temperature exceeds or decreases beyond certain extreme level, the alarm system is triggered. Even though the ideal system should permit autocorrections based on humidity levels, commercially available sensors provide feedback based on changes in temperature [18].  Figure 2 shows an active humidifier with a heated wire in the inspiratory limb; both temperature sensors, one at the side of the patient and the other at the outlet of the heated reservoir, are shown [17]. Usual temperature setting for the current heated humidifiers is 37°C. The performance of humidifiers may be affected by room temperature, as well as patient minute ventilation. In the last situation, an increase in minute ventilation preserving the same temperature of the heated reservoir may not be adequate to deliver appropriate AH to the patient. Therefore, some humidifiers are supplemented with automatic compensation systems, which compute the amount of thermal energy needed to humidify certain volume of gas and change the temperature of the water reservoir accordingly. Lellouche et al. studied the performance of two HWHs and HH devoid of heated wires under different room temperatures (high, 28–30°C; normal, 22–24°C). The authors also investigated device performance by changing the temperature of gas within the ventilators and under two different minute ventilation levels (Ve) (low $\dot{\text{V}}\text{e}$ of 10 L/min and high $\dot{\text{V}}\text{e}$ of 21 L/min). The presence of high minute ventilation and room temperature resulted in a reduction of humidification performance, with absolute humidity of less than 20 mg H2O/L. One of the tested humidifiers had an automatic compensation system for changes in minute ventilation. This model achieved higher AH levels than those that relied only on temperature sensors [19]. Furthermore, other studies have also reinforced the effect of room temperature, variance in minute ventilation, and ventilator gas temperature on levels of absolute humidity delivered to patients [20–22]. Notably, some studies indicate that heated humidifiers without heated wires achieve higher levels of humidification than HWHs. Nevertheless, it is clear that they are associated with more condensation and respiratory secretions [23]. Hence, these types of humidifiers are becoming increasingly unpopular among respiratory care providers. As previously mentioned, inspiratory heated wires can minimize condensation. However, exhaled air can form rainout in the expiratory limb. This has led to the utilization of double heated wire (DHW) circuits. This practice has replaced the use of single heated wires (SHW) circuits in some countries [24]. Another described technique to limit condensate in the expiratory limb is to use porous expiratory circuits [25].

**Figure 3:**
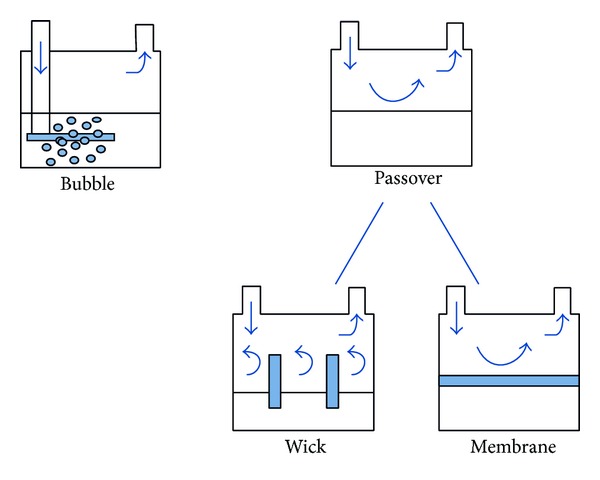
Bubble and passover humidifiers, adapted from* Egan's Fundamentals of Respiratory Care, *10th edition, St. Louis: Mosby-Elsevier; 2012: 1424 [17].

Heated humidifiers have different designs and different techniques for humidification. Accordingly, these devices are classified as (1) bubble; (2) passover; (3) counter-flow; and (4) inline vaporizer.

(*1) Bubble*. In bubble humidifiers, gas is forced down a tube into the bottom of a water container (Figure 3). The gas escapes from the distal end of the tube under water surface forming bubbles, which gain humidity as they rise to the water surface. Some of these humidifiers have a diffuser at the distal end of the tube that breaks gas into smaller bubbles. The smaller the bubbles, the larger the gas-water interface allowing for higher water vapor content. Other factors that influence water vapor content of the produced gas are the amount of water in the container and the flow rate. Simply, the higher the water column in the container, the more gas-water interface will ensue, so water levels should be checked on a frequent basis. In terms of flow rate, when slow flows are delivered, there is more time for gas humidification. Bubble humidifiers may be unheated or heated. Typically, unheated bubble humidifiers are used with low-flow oral-nasal oxygen delivery systems. Heated bubble humidifiers provide higher absolute humidity. They are designed to work with flow rates as high as 100 L/min. These humidifiers usually use diffusers to increase the liquid-air interface. A problem with heated bubble humidifiers is that they exhibit high resistance to airflow imposing higher work of breathing than passover ones [26, 27]. Furthermore, they may generate microaerosol [28, 29]. Nevertheless, the CDC guidelines for prevention of health care associated pneumonia reported that the amount of aerosol produced by these types of humidifiers may not be clinically significant [30]. Despite this statement, the use of bubble humidifiers during mechanical ventilation has fallen in favor of passover ones.

(*2) Passover*. In passover humidifiers (Figure 3), gas passes over a heated water reservoir carrying water vapor to the patient. These are typically used for the purpose of invasive and noninvasive mechanical ventilation. Another variant of passover humidifiers is the wick one (Figure 3). In this type of device, the gas enters a reservoir and passes over a wick that acts as a sponge that has its distal end immersed in water. The wick pores provide more gas-water interface allowing for more humidification compared to simple passover humidifiers. The water reservoir is fed through a closed system. This system can be supplied with water either manually through a port or float feed system that ensures the water level remains constant all the time. As dry gas enters the chamber and travels through the wick, heat and moisture increase. Due to the fact that gas does not emerge underneath the water surface, no bubbles are generated. A third type of passover humidifier involves a hydrophobic membrane (Figure 3). As with the wick device, dry gas passes through a membrane. Nevertheless, its hydrophobic characteristic only allows passage of water vapor, precluding liquid water to travel through it. Similarly to the wick humidifier, bubbles and aerosols are not generated. As mentioned previously, these humidifiers are more commonly used during mechanical ventilation than bubble ones due to their lower flow resistance and absence of microaerosols. In all cases, a temperature probe is placed near the Y piece of the ventilator circuit to ensure delivery of gas with optimal temperature. As it was stated above, the presence of condensate in the tubing may increase resistance, which can decrease volume delivered in pressure controlled, or increase peak pressure in volume controlled modes. Despite the need of the aforementioned heated wires to avoid undesirable condensation, it is also worth mentioning that use of these wires does not come without thermal risks [31]. Consequently, the American Association of Respiratory Care (AARC) clinical practice guidelines recommend gas delivery with a maximum temperature of 37°C and 100% RH (44 mg H2O/L) [11].

In terms of humidifier heating systems, currently there are 6 types of devices. The hot plate element, which sits at the bottom of the humidifier, is one of the most commonly used. Other devices include the wraparound element, which surrounds the humidifier chamber; a collar element, which sits between the reservoir and the outlet; the immersion heater, which is placed directly inside the water reservoir; and the heated wire, which is placed in the inspiratory limb of the ventilator.

(*3) Counter Flow*. In the recently described counter-flow humidifier, water is heated outside the vaporizer. After being heated, water is pumped to the top of the humidifier, enters the inside of the humidifier through small diameter pores, and then runs down a large surface area. Gas flows in counter direction. During its passage through the chamber of the humidifier, the air is moisturized and warmed to body temperature. Schumann et al. compared the counter-flow humidifier, a heated passover, and a heat and moisture exchanger (HME) in an artificial lung model. The authors demonstrated that the counter-flow device imposed less work of breathing compared with the other ones. In addition, the humidification performance of the counter-flow model was independent of flow and respiratory rate, in contrast to the heated passover humidifier in which humidification performance decreased with increasing ventilator rates [32]. This technology is promising but more studies are needed before it becomes widely adapted.

(*4) Inline Vaporizer*. The novel inline vaporizer uses a small plastic capsule where water vapor is injected into the gas in the inspiratory limb of the ventilator circuit immediately proximal to the patient wye. In addition to the water vapor, gas heating is supplemented by a small disk heater in the capsule. Water is delivered to the capsule by a peristaltic pump housed in a controller. The amount of water sent to the capsule is set by the clinician based on minute volume through the circuit. Both temperature and humidity are adjustable and displayed constantly. The proximity to the wye connection obviates the requirement for heated wires and external temperature probes. The manufacturer reports very high AH production with this system. However, this system was only studied during high frequency percussive ventilation [33, 34].

### 3.2. Passive Humidifiers

#### 3.2.1. Heat and Moisture Exchangers (HMEs)

**Figure 4:**
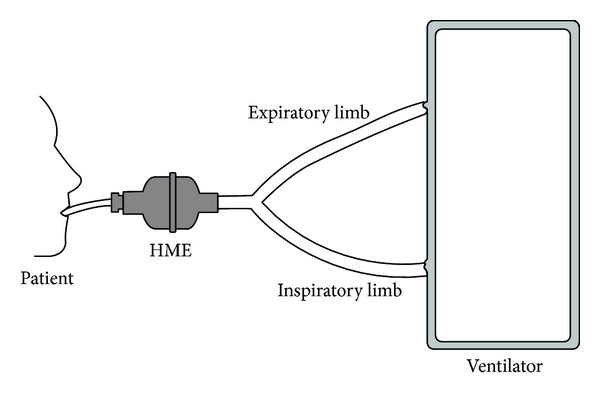
HME position in ventilator circuit.

**Figure 5:**
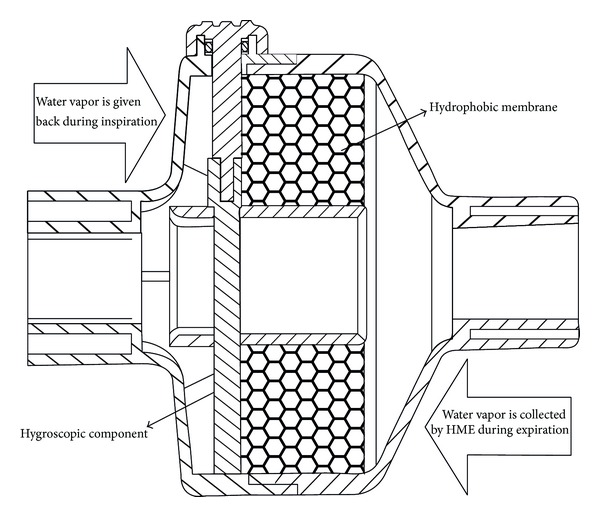
HME.

Heat and moisture exchangers are also called artificial noses because they mimic the action of nasal cavity in gas humidification. They operate on the same physical principle, as they contain a condenser element, which retains moisture from every exhaled breath and returns it back to the next inspired breath. Unlike heat humidifiers, which are placed in the inspiratory limb of the circuit, these devices are placed between the Y piece and the patient (Figure 4). This may increase resistance to airflow not only during inspiration, but also during the expiratory phase. In situations in which administration of aerosolized medications is needed, HMEs need to be removed from the circuit to avoid aerosol deposition in HME filters. Otherwise, HMEs with capability to change from “HME function” to “aerosol function” should be used. Initial designs of HMEs used condensers made of metallic elements that had high thermal conductivity. Thus, they were able to recapture only 50% of the patient's exhaled moisture. Hence, they provided humidification of 10–14 mg H2O/L, at tidal volumes (VT) ranging between 500 mL and1000 mL. These devices were known as simple HMEs. They were not disposal and created a significant resistance during mechanical ventilation [35, 36]. Newer designs of HMEs include hydrophobic, combined hydrophobic hygroscopic, and pure hygroscopic HMEs. In hydrophobic HMEs, the condenser is made of a water repelling element with low thermal conductivity that maintains higher temperature gradients than in the case of simple HMEs. In combined hydrophobic hygroscopic HMEs, a hygroscopic salt (calcium or lithium chloride) is added inside the hydrophobic HME. These salts have a chemical affinity to attract water particles and thus increase the humidification capacity of the HME. Pure hygroscopic HMEs have only the hygroscopic compartment. During exhalation, vapor condenses in the element as well as in the hygroscopic salts. During inspiration, water vapor is obtained from the salts, obtaining an absolute humidity ranging between 22 and 34 mg H2O/L.  Figure 5 illustrates the basic structure and work principle of HMEs.

Hydrophobic HMEs were found to cause more narrowing in ETT diameter compared to hygroscopic ones [37]. Therefore, the aforementioned HMEs are not frequently used. Filters can be added to either hydrophobic or hygroscopic HMEs resulting in a heat and moisture exchanging filter (HMEF). These filters operate based on electrostatic or mechanical filtration. Specifically, based on the predominant mechanism applied, these filters may be classified into pleated or electrostatic filters. The pleated filters have more dense fibers and less electrostatic charges, whereas the electrostatic filters have more electrostatic charges and less dense fibers. Pleated filters function better as barriers to bacterial and viral pathogens than electrostatic filters. However, they confer higher airflow resistance [38]. The pleated nature of the membrane causes a turbulent air flow, which increases the pathogen's deposition onto the inside of the filter. The electrostatic filters are subjected to an electric field. Since bacteria and viruses carry electric charges, they get trapped within the electric field of these filters. These filters usually have larger pores than the pleated membranes, and they rely mainly on the electrostatic mechanism. The previously described filter confers little to the humidification process and increases resistance. Therefore, they are mainly used as barriers to pathogens [15]. HMEs design and performance standards are defined by the International Organization for Standardization (ISO). According to these standards, the appropriate HME should have at least 70% efficiency, providing at least 30 mg/L of water vapor. In a recent study, Lellouche and colleagues independently assessed the humidification capacity of 32 HMEs. Strikingly, 36% of tested HMEs had an AH of 4 mg H2O/L lower than what was listed by the manufacturer. In fact, in some of them the difference was higher than 8 mg H2O/L [39].

Intuitively, as HMEs eliminate the problem of tubing condensation, it may be considered as “elements of choice” to prevent ventilator-associated pneumonia (VAP). Nevertheless, whether the presence of tubing condensate represents an important factor for the development of VAP in well-maintained circuits remains controversial. Furthermore, HMEs also present some shortcomings. Specifically, impaction of secretions or blood within the device may increase airway resistance and work of breathing. In extreme circumstances, complete airway obstruction has been reported [40]. Therefore, patient selection becomes an essential component in the use of HMEs.  Table 2 shows contraindications for the use of HMEs [11].

In certain devices, an active heated water source can be added to HMEs converting them from passive to active, increasing their humidification capacity. If the external source of water runs out, these devices will still work as passive HMEs. Several models exist, including the Booster, the Performer, the Humid Heat, and the Hygrovent Gold.

In the Booster model, the heating unit is incorporated between the HME and the patient. During inspiration the gas passes through the HME carrying water vapor based on the passive operation of the HME and then the heating unit adds to the humidity content of the gas before it reaches the patient. As water enters the HME-Booster, it saturates the hydrophobic membrane contained in it. The moisture in the saturated membrane is then heated by the positive temperature control element connected to it [41]. It is thought that the utilization of this device may increase AH by 2-3 mg/L of H2O more than passive HMEs [42].

**Figure 6:**
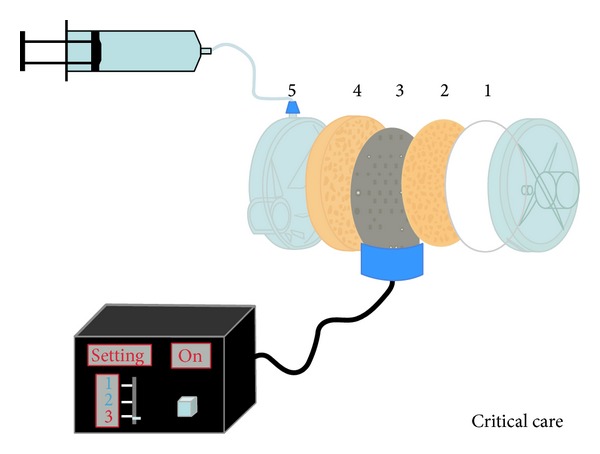
HME-Performer. Adapted from Critical Care, with permission [42].

The Performer device is characterized by a metal plate in the middle of the HME, in between two hydrophobic and hygroscopic membranes (Figure 6). This metal plate is heated by an external source that has three sets of temperature to deliver 40°C, 50°C, and 60°C. A water source provides it to one end of the humidifier. The water reaches the two membranes and the metal plate heats it. Then, the water evaporates augmenting vapor content in the inspired gas. The performer is able to deliver AH of 31.9 to 34.3 under normothermic conditions [42].

The Humid Heat is a hygroscopic HME that has an external heating source with the water being added at the patient side [15]. In one bench study, it was found to provide an absolute humidity of 34.5 mg H2O/L [43]. Humid Heat has preset values for temperature and humidity. The only parameter that needs to be set is the value of minute volume of the ventilator, making its use very simple.

The Hygrovent Gold is an active hydrophobic HME that has an adapter to which a heating element can be inserted and a water line to supply water inside the HME. There is a thermal sensor to avoid overhumidification. Under normothermic conditions, it was reported to provide an AH of 36.3 mg H2O/L. Increased flow resistance can be found with these active humidifiers, which is likely related to accumulation of water condensate in the passive component [44].

Last, another active HME model is based on chemical reactions. In these HMEs, the carbon dioxide in the exhaled breath is exploited to generate heat through a chemical reaction when it passes through the humidifier. Broach and Durbin Jr. conducted a randomized controlled clinical trial on fifty patients undergoing coronary artery bypass grafting and compared between chemically heated HME and the conventional passive ones. The chemically heated HME resulted in more rapid rewarming of mildly hypothermic patients, with no difference in clinical outcomes [45]. Due to limited experience with this device, chemically active HMEs are not currently used in clinical practice.

## 4. Monitoring of Humidification Systems

Upon setting humidification levels in mechanically ventilated patients, respiratory therapists usually follow the American National Standard Institute (ANSI) recommendations, which involve a level of water vapor that exceeds 30 mg/L. In fact, recent guidelines published by the American Association of Respiratory Care (AARC) recommend a temperature of 33 ± 2°C with RH of 100% and a water vapor level of 44 mg/L. Despite the aforementioned guidelines, the clinician commonly faces the issue of relying on different humidifiers without being certain about device accuracy. Independent assessments raise concerns about the validity of data included by the manufacturer [39]. The most reliable mean to measure humidity is by using a hygrometer-thermometer system. However, these devices are not always available at the bedside for every patient. Consequently, different surrogate markers have been suggested to monitor humidification levels. The most popular surrogates are secretion characteristics, visual observation of condensate in tubing system, and requirement for saline instillation. In general, the volume of secretions is directly proportional to degree of humidification. Excessive humidification will increase secretion volume, and suboptimal humidification will lead to crusting, inspissation of secretions, and a decrease in their volume [46]. Nevertheless, this relationship assumes that humidity is the only factor that influences secretion volume. As a matter of fact, secretion volume may be altered by administered aerosolized medications, frequency of suctioning, and saline instillation [47]. Frequency of saline instillation has been proposed by some as a surrogate of gas humidity. However, this practice can tremendously differ from one practitioner to another [48]. Ricard and colleagues conducted a prospective randomized clinical trial on 45 mechanically ventilated patients to assess whether visual observation of condensate in the tubing system would correlate with hygrometric studies of HMEs and HHs. An independent observer unaware of the hygrometric results rated condensate in the tubing system as follows: dry, moisture only, moisture plus few water droplets, moisture plus several water droplets, moisture plus numerous water droplets, and dripping wet. Interestingly, there was a significant correlation between the visual observation method and the hygroscopic measurements [49]. Despite the previously described data, there is still no clear consensus about a universal way to assess for humidity adequacy at the bedside.

## 5. Selecting the Appropriate Humidifier

### 5.1. Humidification Performance

According to AARC guidelines, HHs should provide an absolute humidity level between 33 and 44 mg H2O/L, whereas HMEs should provide a minimum of 30 mg H2O/L [11]. Initial studies testing HMEs addressed their performance in the anesthesia settings, which entailed testing them for short periods of time. In a bench study, six different HMEs were found to provide an AH as low as 14 to 26 mg H2O/L [50]. As HMEs started to be tested in the intensive care unit setting, concerns regarding increased incidence of ETT occlusions aroused. In a case series, Cohen et al. reported 15 cases of ETT occlusion when a hydrophobic HMEF was used, whereas only one case with bubble humidifiers was demonstrated. Nevertheless, most patients with ETT occlusion required minute ventilations higher than 10 L/min, reducing the generalizability of these results [51]. In a prospective randomized controlled trial, an HMEF was compared to HHs. The HMEF was exchanged daily. Data was analyzed from 31 patients in the HMEF and 42 patients in the HH group. Six patients in the HMEF group had occluded ETT, whereas no occlusion was noted in the HH group [38]. The study was prematurely terminated after the death of a patient with complete obstruction of his tracheal tube. Also, Roustan et al. found more ETT occlusions with an HMEF, when it was compared to a HH [52]. However, it is worth noting that these studies were performed with hydrophobic HMEs, and most ETT occlusions were reported with high minute ventilation. Based on the aforementioned information, combined hydrophobic hygroscopic HMEs should be the first choice if passive humidification is selected, as they have better humidification capacity than the hydrophobic ones [53–55]. In fact, a randomized controlled trial comparing hydrophobic hygroscopic HME versus hydrophobic HME versus HH and with minute ventilations of 10.8 L/min, 11.6 L/min, and 10.2 L/min showed that, after 72 hours, the mean diameter of the ETTs had decreased by 6.5 mm with the hydrophobic HME, 2.5 mm with hygroscopic hydrophobic HME, and 1.5 mm with an HH [37]. In a multicenter randomized controlled prospective trial, patients expected to require mechanical ventilation for more than 48 hours were randomly assigned to either a combined hydrophobic hygroscopic HMEF or a HWH. Endotracheal tube occlusion occurred in five patients in the HWH and in only one patient in the HMEF group. However, this difference was not statistically significant. Of note, patients with contraindications for HMEs were excluded from this trial, mostly due to presence of thick secretions [56].

In terms of HME length of use, some concerns of decreased performance with their prolonged duration have been expressed. Hence, most manufacturers recommend exchanging HMEs every 24 hours. This issue has been an area of evolving research. Djedaini et al. demonstrated that there was no increase in the resistance of hygroscopic hydrophobic HMEs if they were changed every 48 hours versus every 24 hours [57]. Another study revealed that hygroscopic hydrophobic HMEs attained similar absolute humidity levels when used for 24 or 48 hours, with no increase in mean airway pressures at 48 hours [58]. Similar results were demonstrated in subsequent studies using HMEs for 48 hours instead of 24 hours [59, 60]. Furthermore, a study showed that HMEs could be used for 96 hours without significant change in their production of absolute humidity. Nevertheless, this data was obtained from a group of only 13 patients who were mechanically ventilated for neurologic reasons, without prior history of chronic respiratory problems [61]. In a nonblinded prospective randomized controlled study, Thomachot et al. tested the extended use of hydrophobic HMEs for 7 days. Notably, there were no incidents of ETT occlusions, and resistance of HMEs was not increased compared with exchanging them every 24 hours [62]. Last, Kapadia et al. conducted a study to record airway accidents in more than 7900 mechanically ventilated patients over 6 years. In the initial 3 years of the study, HMEFs were changed every 24 hours, and this period was associated with no episodes of tracheal tube occlusion. In the last 3 years of the study, HMEFs were changed every 48 hours, and this was associated with 13 tracheal tube occlusions out of 2932 subjects [63]. This incidence of tracheal tube occlusions would still be very low compared to the studies done on the poorly performing hydrophobic HMEs [51–53].

It is worth mentioning that, as HMEs are passive devices that require retention of heat to provide effective function, they are deemed contraindicated for hypothermic patients with temperatures lower than 32°C [11]. In fact, Lellouche and colleagues conducted a prospective randomized crossover trial to examine the effect of HMEs in nine patients with moderate hypothermia after cardiac arrest. HMEs lead to underhumidification compared to heated humidifiers [64]. In order to compensate for this potential disadvantage, active HMEs were incorporated to clinical practice. Despite eventual benefits in humidification, they have the disadvantage of placing a heat source near the patient, and their use entails a higher dead space than passive HMEs [65]. Also, HMEs are associated with increased risk of ETT occlusion compared to heated humidifiers. Thus, it is advisable not to be used in patients with viscid secretions [66].

### 5.2. Effect on Ventilatory Mechanics

HMEs have unfavorable effects on ventilation parameters. They increase the dead space, which in turn decreases alveolar ventilation and leads to increase in arterial carbon dioxide tension. Hence, in order to keep the same level of alveolar ventilation, tidal volume has to be increased exposing patients to volume-induced lung injury. In spontaneously breathing patients, the addition of death space associated with HMEs may increase work of breathing precluding liberation from mechanical ventilation [67]. Prat and colleagues demonstrated a mean of 17 mm Hg decrease in PaCO2 levels in ARDS patients, when heated humidifiers were used instead of HME. This was thought to be related to a difference in dead space of 95 mL between devices [68]. Optimization of the PaCO2 in ARDS patients by means of replacing HMEs for HHs was also demonstrated in other studies [69–71]. Le Bourdellès et al. conducted a randomized crossover trial comparing HME to HH during weaning of fifteen patients. They suggested that although dead space added by HMEs may be trivial, it may adversely affect weaning process in patients with limited respiratory reserve [72]. This finding was subsequently reinforced by a later prospective randomized controlled study done by Girault and colleagues on eleven mechanically ventilated patients with chronic respiratory failure [73]. Furthermore, Iotti and colleagues compared the effects of a HH, HME without a filter, and HMEF on ten patients ventilated with PSV mode. The highest increase in dead space and dynamic hyperinflation were seen with the HMEF. This was revealed by an increase in required pressure support, which ranged from means of 12.8 cm H2O with HHs, 14.8 cm H2O with HMEs without filter, and 17.6 cm H2O with HMEFs [74]. In addition to the dead space effect, HMEs increased inspiratory and expiratory resistance, which contributed to the development of intrinsic PEEP [75].

### 5.3. Association with Ventilation Associated Pneumonia (VAP)

In 1998, Cook et al. conducted a meta-analysis that included five randomized controlled studies performed between 1990 and 1997. The authors found lower VAP rates with the use of HMEs compared with heated humidifiers [76]. However, these lower rates of VAP were mostly found in only one among five included studies [77]. In a subsequent meta-analysis, no difference was found in VAP rates between HH and HMEs [78]. The most recently published meta-analysis included thirteen randomized controlled studies. It found no difference in the incidence of VAP [79]. The difference in results between these meta-analyses can be attributed to the diversity of studies included. Moreover, these studies included different types and designs of HMEs and HHs. This heterogeneity was reflected on guidelines proposed by different societies. In the guidelines published in 2008 by the British Society for Antimicrobial Chemotherapy, it recommended the use of HMEs over HHs to reduce the incidence of VAP [80]. Nevertheless, this guideline did not include the results of the meta-analysis performed by Siempos and colleagues in 2007, which included the largest number of trials among four meta-analyses performed to date. This meta-analysis found no difference in the incidence of VAP between HMEs and HHs. The CDC recommendations did not favor HMEs over HHs [81], and the American Thoracic Society stated that HMEs cannot be regarded as a tool for prevention of VAP [82]. In 2009, the European Respiratory Society (ERS), the European Society of Clinical Microbiology and Infectious Diseases (ESCMID), and the European Society of Intensive Care Medicine (ESICM) issued a joint statement preferring HMEs over HHs for the prevention of VAP. However, this was solely based on the work of Torres et al. without including subsequent studies and meta-analyses [83]. In the same year, the VAP Guidelines Committee and the Canadian Critical Care Trials Group stated that there was no difference in the incidence of VAP between HMEs and HHs [84]. The inclination of the European guidelines toward HMES coincides with the trend in clinical practice. A cross-sectional survey denoted that HMES were more commonly used in France than in Canada [85].

In brief, based on the previously described data, humidifier selection should be made according to the specific clinical context. In general, HMEs are easy to use and lighter than heated humidifiers. Therefore, they facilitate transportation of mechanically ventilated patients, and they do not carry the same thermal hazards. Theoretically, heated humidifiers confer better humidity than HMEs. They are generally preferred in patients with viscid secretions or when long term ventilation is sought. However, in a recent Cochrane systematic review, there was no difference in clinical outcomes. Yet, in the same review, Paco2 and minute ventilation were found to be higher with HMEs suggesting that heated humidifiers could be better options in patients with limited respiratory reserve [86]. A characteristic disadvantage of heated humidifiers is the formation of condensate in the circuit, which was associated in earlier studies with an increased risk of nosocomial infections [77]. Despite the previously described finding, no difference was found in pneumonia rates between heated and passive humidifiers [86].

## 6. Summary

Airway humidification represents a key intervention in mechanically ventilated patients. Inappropriate humidifier settings or selection of devices may negatively impact clinical outcomes by damaging airway mucosa, prolonging mechanical ventilation, or increasing work of breathing. Humidifier devices may function passively or actively, depending on the source of heat and humidity. Depending on the clinical scenario, humidifier selection may change over time. Therefore, knowledge of the advantages and disadvantages of each of these devices is essential for respiratory care practitioners.

## Figures and Tables

**Table 1:** Humidity requirements for gas delivery at different anatomic sites in the airway.

Anatomic site	Nose or mouth	Hypopharynx	Midtrachea
Humidity requirements	50% relative humidity with AH of 10 mg/L at 22°C	95% RH with AH of 28 to 34 mg/L at 29 to 32°C	100% RH with AH of 36 to 40 mg/L at 31 to 35°C

Adapted from Cairo [12].

**Table 2:** Contraindications for heat and moisture exchangers according to AARC Clinical Practice Guidelines 2012 [11].

(i) Patients with thick or copious secretions.

(ii) When there is loss in expired tidal volume (e.g., large bronchopleurocutaneous fistulas or presence of endotracheal tube cuff leak).

(iii) In patients managed with low tidal volumes like those with ARDS.

(iv) In difficult to wean patients and those with limited respiratory reserve.

(v) Hypothermic patients with body temperature of <32°C.

(vi) In patients with high minute ventilations volumes (>10 L/min).
